# Highly Conductive Aromatic Functionalized Multi-Walled Carbon Nanotube for Inkjet Printable High Performance Supercapacitor Electrodes

**DOI:** 10.1371/journal.pone.0131475

**Published:** 2015-07-08

**Authors:** Sanjeev K. Ujjain, Rohit Bhatia, Preety Ahuja, Pankaj Attri

**Affiliations:** 1 Department of Physics, Indian Institute of Technology Kanpur, Kanpur, UP, India; 2 Department Of Chemistry, Institute Of Home Economics, Delhi, India; 3 Peoples Action For Livelihood & Sustainability (Pals), Delhi, India; 4 Department of Chemistry, University of Delhi, Delhi, India; 5 Plasma Bioscience Research Center / Department of Electrical and Biological Physics, Kwangwoon University, Seoul, Korea; US Naval Reseach Laboratory, UNITED STATES

## Abstract

We report the functionalization of multiwalled carbon nanotubes (MWCNT) via the 1,3-dipolar [3+2] cycloaddition of aromatic azides, which resulted in a detangled CNT as shown by transmission electron microscopy (TEM). Carboxylic moieties (-COOH) on aromatic azide result in highly stable aqueous dispersion (max. conc. ~ 10 mg/mL H_2_O), making the suitable for inkjet printing. Printed patterns on polyethylene terephthalate (PET) flexible substrate exhibit low sheet resistivity ~65 Ω. cm, which is attributed to enhanced conductivity. Fabricated Supercapacitors (SC) assembled using these printed substrates exhibit good electrochemical performance in organic as well as aqueous electrolytes. High energy and power density (57.8 Wh/kg and 0.85 kW/kg) in 1M H_2_SO_4_ aqueous electrolyte demonstrate the excellent performance of the proposed supercapacitor. Capacitive retention varies from ~85–94% with columbic efficiency ~95% after 1000 charge/discharge cycles in different electrolytes, demonstrating the excellent potential of the device for futuristic power applications.

## Introduction

Printed electronics including light-emitting diodes, solar cells, and printable transistors, have the potential to achieve an ever increasing demand for next generation high energy and power sources in miniature form [1]. However, to provide the required peak power, conventional charge storage devices such as batteries are bulky and heavy. Consequently, batteries are not suitable for future generation portable electronic devices [2]. Electrochemical supercapacitors having high power with excellent cycling stability are considered as the most promising complementary energy source to fulfill future energy storage demands [3–6]. Electrochemical supercapacitors are divided into two categories based on their charge storage mechanisms: (a) Electrical double layer capacitors (EDLCs), in which charge accumulation occurs at the electrode/electrolyte interface and (b) Pseudocapacitors that employ faradic redox transitions. Carbonaceous materials such as activated carbons, aerogels, carbon fibers, and other nanostructured carbon materials store charge by EDL formation, while redox transitions are responsible for the charge storage in transition metal oxides and conducting polymers [3,7].

Among the different carbon materials [8–10], carbon nanotubes (CNT) are an attractive component of supercapacitors due to their excellent thermal conductivity [11], good mechanical strength [12], high surface area, uniform pore size distribution [13], and elective semiconducting/metallic nature [14]. For example, Kaempgen *et al*. [1] employed CNT film serving both as electrodes and charge collectors with very high power densities of 23 and 70 kW/kg for aqueous gel and organic electrolyte, respectively. Heat-treated arc discharge SWNT demonstrate an energy density of 7 Wh/kg and power density of 20 kW/kg in the potassium hydroxide (KOH) electrolyte [15]. However, the strong intertube van der Waals and π-π interactions constrain their practical application [16]. In order to increase its processability, carboxylation by high temperature acid treatment and additional dispersants are extensively used [2,17–19]. The carboxylation method disrupts the sp^2^ carbon network of nanotubes, while the dispersants increase the resistance by creating a physical barrier around the CNT, inhibiting the efficient contact of the, rendering them unsuitable for electronic applications [20].

In this work, we focused on the 1,3-dipolar [3+2] cycloaddition of azides with CNT. The presence of nitrogen influences the electronic properties of nanotubes and hence the device performance [21]. Based on our previous studies [22], we developed a convenient approach towards the azide functionalization of MWCNT for the solubilisation of the MWCNT in polar as well as non-polar solvents. The presence of carboxylic moieties (-COOH) on azide resulted in highly stable aqueous dispersion (max. concentration _**~**_ 10 mg/mL H_2_O), making azide suitable for inkjet printing. The successful inkjet printing of *f*-MWCNT on PET or flexible (ITO) substrates is demonstrated. The morphology and electrical properties of *f*-MWCNT are analyzed and successfully investigated for use as a supercapacitor electrode material.

## Materials and Methods

### Materials

Chemical reagents of AR grade were used for the experimental work. Ortho dichlorobenzene (ODCB), chlorobenzene, acetonitrile (ACN), isopropanol (IPA), ethanol, dimethyl formamide (DMF), chloroform, sulphuric acid and metal salts, KOH, and sodium sulphate (Na_**2**_SO_**4**_) were purchased from Merck. (TBAP) was procured from Sigma Aldrich, USA. Millipore water was used for preparation of the aqueous solutions and for rinsing.

### Characterization

TEM measurements were performed with a Philips Technai G^2^30 microscope operating at an accelerating voltage of 300kV. Surface morphological characterization of the *f*-MWCNT inkjet printed electrode on the PET substrate was performed with a Zeiss Ultra 55 field emission scanning electron microscope (FESEM). A Renishaw inVia Raman microscope equipped with a laser having a wavelength of 514 nm was used for Raman analysis of *f*-MWCNT. Fourier transform infrared (FTIR) spectra were recorded on a Perkin-Elmer FT-IR spectrum BX spectrometer in the range of 4000–400 cm^-1^. Thermogravimetric analysis was carried out on a Perkin-Elmer Diamond TG/DTA instrument in air atmosphere from 25 to 900°C.

Electrochemical analyses were evaluated using a piece of platinum gauze and Ag/AgCl as the counter and reference electrode respectively. *f*-MWCNT film on ITO substrate acted as working electrode. The supercapacitor (SCs) cell performances were studied using Electrochemical impedance analysis (EIS) and Cyclic voltammetry (CV) on electrochemical analyzer (Model: CHI 604 D, CH Instruments, USA), and Galvanostatic charge–discharge (GCD) were studies using Arbin Instruments, USA (model: BT 2000). The specific capacitance was calculated using the following equations [23].

$$C=\frac{I}{\nu m}$$

where I is the current, υ is the scan rate, and m is the mass loading on two electrodes.

The energy density (E in Wh kg^-1^) and power density (P in kW kg^-1^) were calculated from the galvanostatic charge/discharge curve using the following expressions.

$$E=\frac{1}{2\times 3.6}CV^{2}$$

$$P=\frac{V^{2}}{4mR}$$

where C is the measured device capacitance, V is the operating voltage window, m is the loading of active material on two electrodes of the cell, and R is the equivalent series resistance calculated from the IR drop in the Galvanostatic discharge curve.

### Functionalization of Carbon Nanotubes

In order to attain improved functionalization, pre-treatment of MWCNT before covalent modification under microwave irradiation was performed. 10 mg of the dried material was inserted into the vial and subjected to microwave irradiation in a domestic microwave oven at 100 W for 15 min with a 3 min on and 1 min off cycle. For covalent functionalization reaction, 10 mg of microwave treated MWCNT was suspended in a three-neck flask with 10 ml of ortho dichlorobenzene as a solvent and sonicated for six hours. A reaction vessel was then placed in a pre-heated oil bath at 180°C. In another test tube, 110 mg of aromatic azide (synthesized using our previously reported process)^22^ was dissolved in 5 ml of chlorobenzene and, using a syringe, was added slowly to the reaction vessel over a period of 1 hour. An inert atmosphere was maintained throughout using nitrogen. The progress of the reaction was monitored using TLC plates for checking the decomposition of the azide. After 4 hours, no azide could be detected and the reaction was quenched by removing it from the heat. The reaction mixture was then allowed to cool to room temperature and subjected to centrifugation to remove the unfunctionalized carbon nanotubes. The supernatant was collected and the product was precipitated using acetone. The precipitated product was obtained by centrifugation with repeated washing with millipore water and the final product (3 mg) was dried in an oven at 70°C.

### Inkjet printing of *f*-MWCNT for supercapacitor electrode

*f*-MWCNT was dispersed ultrasonically in distilled water to prepare the CNT ink (CNT concentration is 3 mg/ml). The black ink cartridge of an inkjet office printer (HP Deskjet D2360) was removed, and the cartridge was thoroughly washed with IPA and water, and then dried in air. The *f*-MWCNT ink prepared as described above was then loaded in the cartridge. The CNT films (1x8 cm) were printed on PET and flexible ITO substrates. For SEM and resistivity measurements, *f*-MWCNT film on PET substrate was used while for electrochemical measurements, strips (1x2 cm) were cut from the printed ITO film. A supercapacitor cell was then assembled by installing Nafion 115 membrane as a separator between the two similar electrodes.

## Results and Discussion

**Fig 1:**
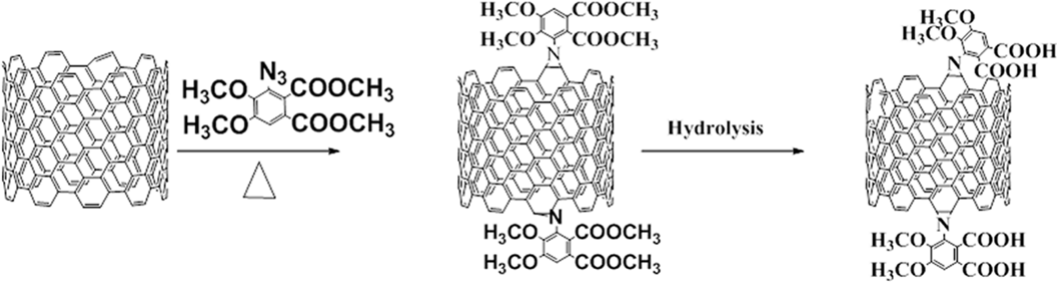
Functionalization of MWCNT with aryl azide.

**Fig 2:**
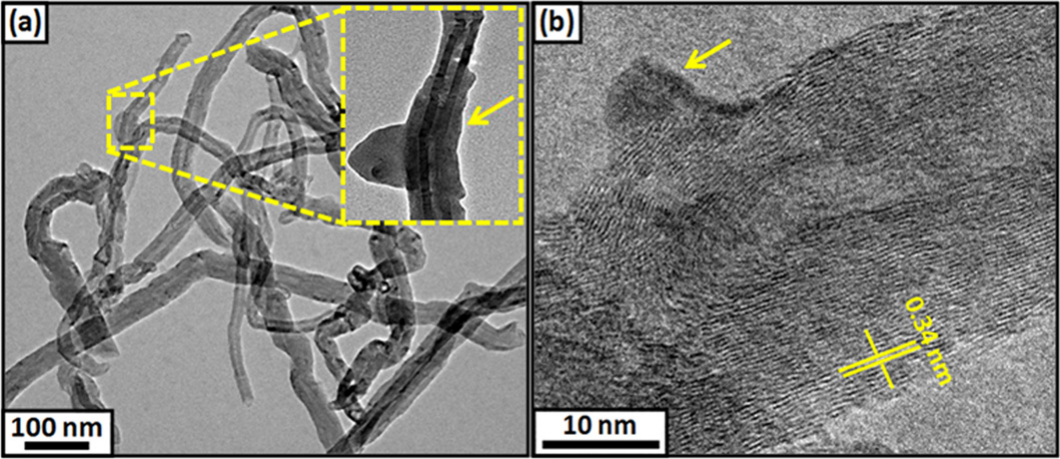
(a) TEM of *f*-MWCNTs; inset shows magnified image with change in morphology of MWCNT on functionalization and (b) HRTEM with fringe width 0.34 nm. Arrow heads demonstrate presence of adduct.

Fig 1 shows the schematic pathway for modification of MWCNT. It shows that the 1,3-dipolar [3+2] cycloaddition of azides to MWCNT sidewalls followed by thermal extrusion of N_2_ from the triazoline intermediate resulted in aziridino functionalized MWCNT (*f*-MWCNT). This approach of covalent functionalization is particularly important, as cycloaddition carried out according to the described conditions does not become physically damage or break the CNT structure, thus retaining its high conductivity [24]. The efficient functionalization of MWCNT was observed using TEM (Fig 2a), showing highly dispersed CNT. After functionalization, the strong van der Waal forces of attraction between the tubes were disturbed, resulting in the breaking of super bundles and consequent enhanced solubility in common organic solvents. The inset figure demonstrates the change in morphology of the carbon nanotubes after functionalization, shown with the yellow arrow, resulting from the high degree of functionalization. The HRTEM image (Fig 2b) shows clear fringes with a d spacing of 0.34 nm and the diameter of *f*-MWCNT is found to be ~30 nm. To identify the features that change through azide functionalization on MWCNT, the pristine MWCNT and *f*-MWCNT samples are further studied using Raman spectroscopy.

**Fig 3:**
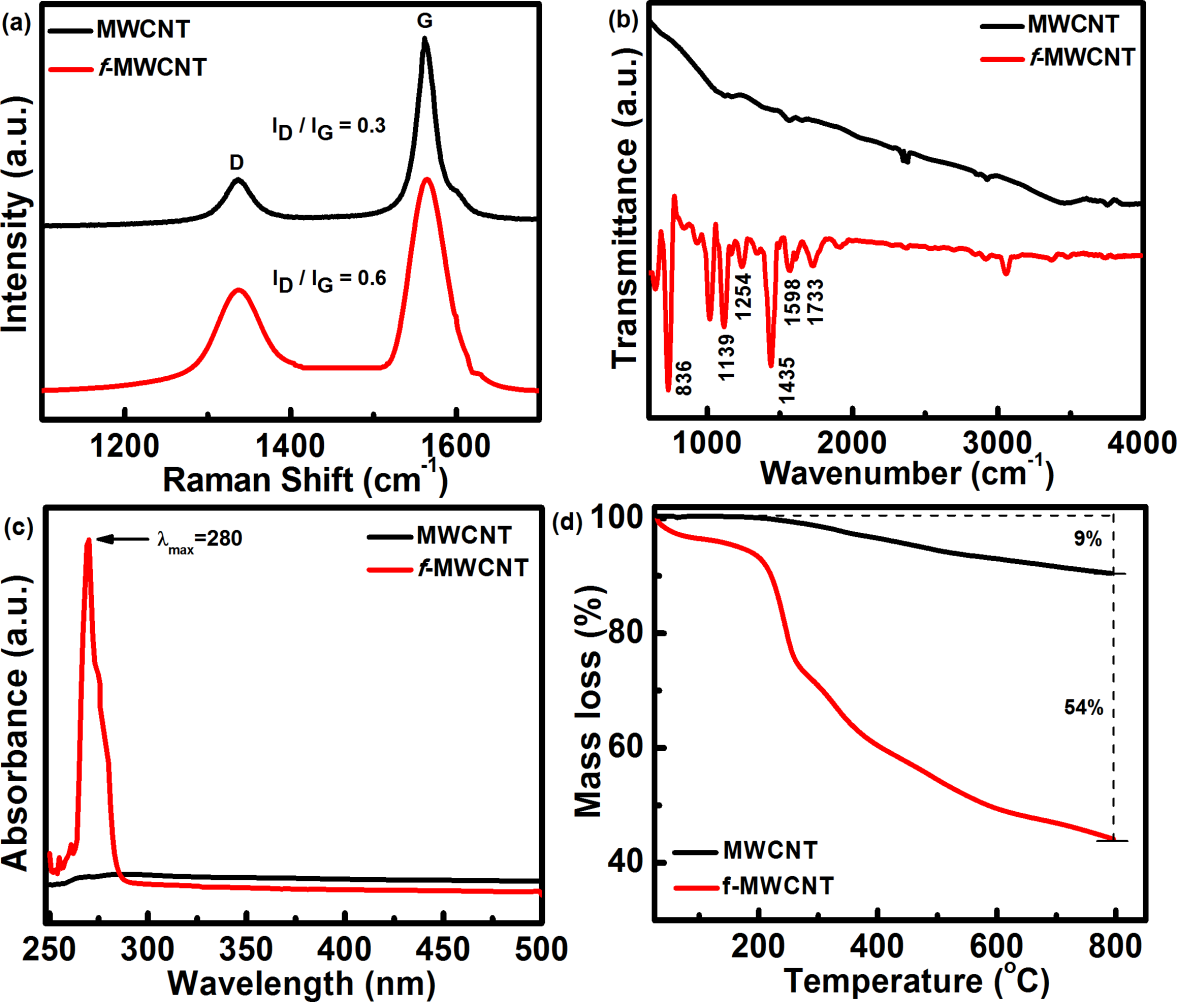
(a) Raman spectra, (b) FTIR spectra, (c) UV-Vis spectra, and (d) Thermogravimetric curves of MWCNT and *f*-MWCNT.

**Fig 4:**
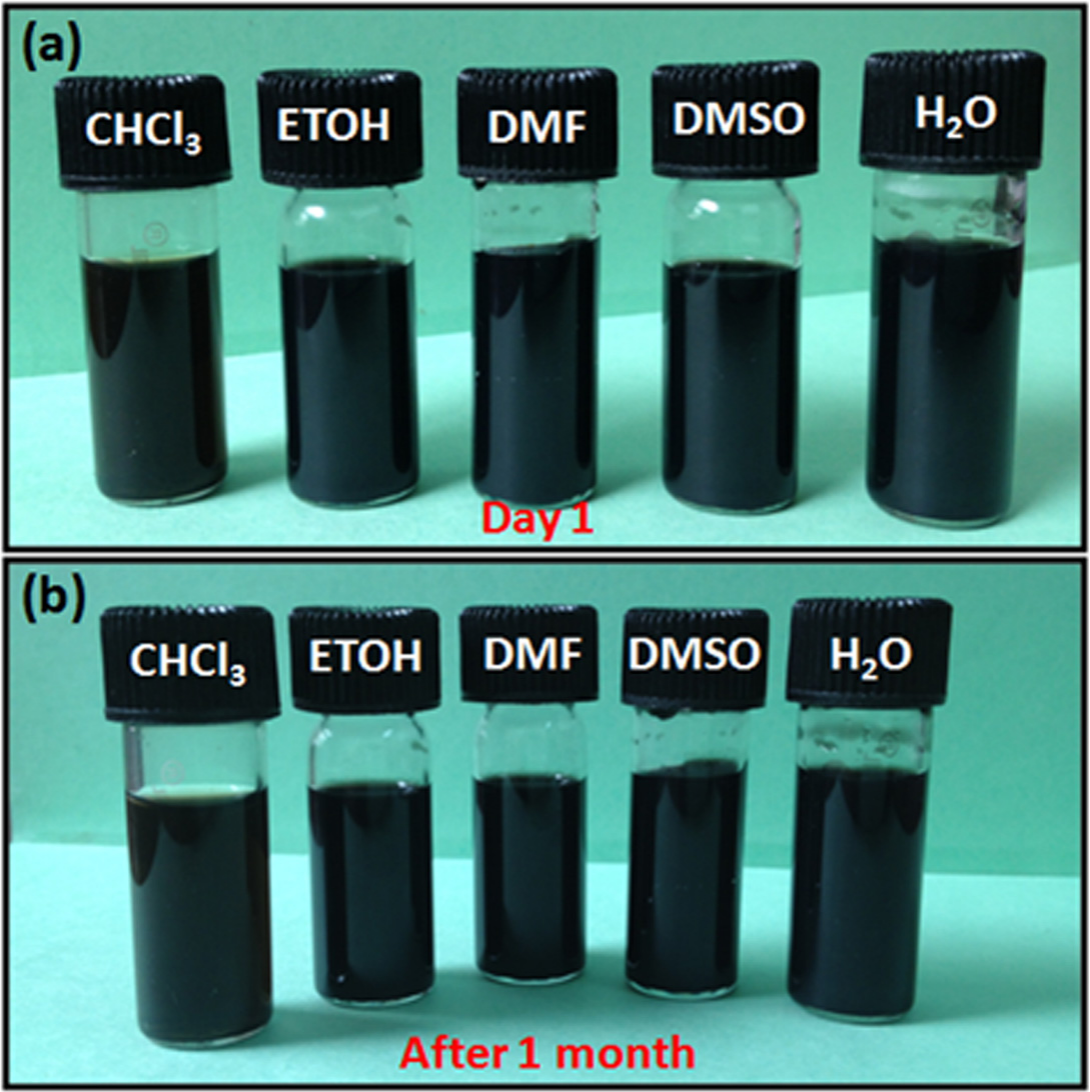
Photographic image of *f*-MWCNT dispersion in chloroform, ethanol, DMF, DMSO, and H_2_O (a) before 1 month and (b) after 1 month.

Covalent functionalization significantly alters the Raman spectrum of pristine MWCNT and *f*-MWCNT, as shown in Fig 3a. Pristine MWCNTs have D and G bands, observed at 1336 cm^-1^ and 1561 cm^-1^, respectively, with an intensity ratio (I_D_/I_G_) of ~0.30. After functionalization, due to insertion reaction, a large amount of the sp^2^ carbon is converted to sp^3^, leading to an increment in the D band intensity. *f*-MWCNT shows increased I_D_/I_G_ to 0.60 after functionalization, demonstrating the presence of the sp^3^ carbon network after functionalization [19]. The presence of different functionalities and the quantitative determination of aryl azide content in *f*-MWCNT was explored by FTIR, UV-Vis, and TGA, respectively. Fig 3b shows the FTIR spectra of pristine MWCNT and *f*-MWCNT. It is well known that MWCNT does not absorb a great deal of in the infrared region. In case of *f*-MWCNT, two important peaks were monitored: (1) the peak around the 2100 cm^-1^ for the azide group and (2) the peak for the carbonyl in the region of 1700–1800 cm^-1^. An absence of peak at 2100 cm^-1^ is indicative of the complete conversion of the azide into nitrene, which then attaches to the side wall of the carbon nanotubes [25]. Further, the characteristic peak observed at 1733 cm^-1^ is assigned to the carbonyl functional group which is present in the aryl azide unit. Peaks at 1254 and 1139 cm^-1^ can be assigned to C-O stretch and out of plane deformation, respectively. The strong peak observed at 1598 cm^-1^ is attributed to the C = C stretching of the benzene ring with C-H bending from the peak at 836 cm^-1^ [26,27] The UV-Vis spectrum (Fig 3c) of pristine MWCNT does not show any well defined peaks; however, background absorption is observed due to the presence of bundles of carbon nanotubes. In comparison, the UV-Vis spectrum of *f*-MWCNT shows an intense peak at 280 nm due to the n-π* transition in the aromatic chromophore [28]. The high intensity of the peak can be assigned to the large number of chromophores present on the carbon nanotube. The degree of functionalization on MWCNT is quantitatively determined by TGA (Fig 3d). The pristine MWCNT demonstrates an overall mass loss of ~9% at up to 800°C under N_2_ atmosphere, which is attributed to the organic and inorganic impurities (including organic solvents) trapped in the MWCNTs. In the case of *f*-MWCNTs, the first major loss between 100°C to 300°C can be attributed to the organic group attached to the side wall of the carbon nanotubes. Mass loss from 300°C to 600°C may be due to the carbonyl groups present on the aryl functional group attached to the side wall. No significant mass loss was observed after 600°C, with a total mass loss of ~54%, indicating a high level of functionalization. Consequently, *f*-MWCNT demonstrates high solubility in both non-polar and polar solvents. Fig 4 shows a digital photograph of the *f*-MWCNT dispersion in chloroform, ethanol, DMF, DMSO, and H_2_O before and after 1 month. The dispersions showed no change even after 1 month. *f*-MWCNTs form stable dispersion on mild sonication up to a maximum concentration of ~10 mg/ml in aqueous medium, which is found to be much higher than that of an existing report for functionalized CNT [20]. This dispersion was then used as ink for the inkjet printer to form *f*-MWCNTs film on PET and a flexible ITO substrate.

**Fig 5:**
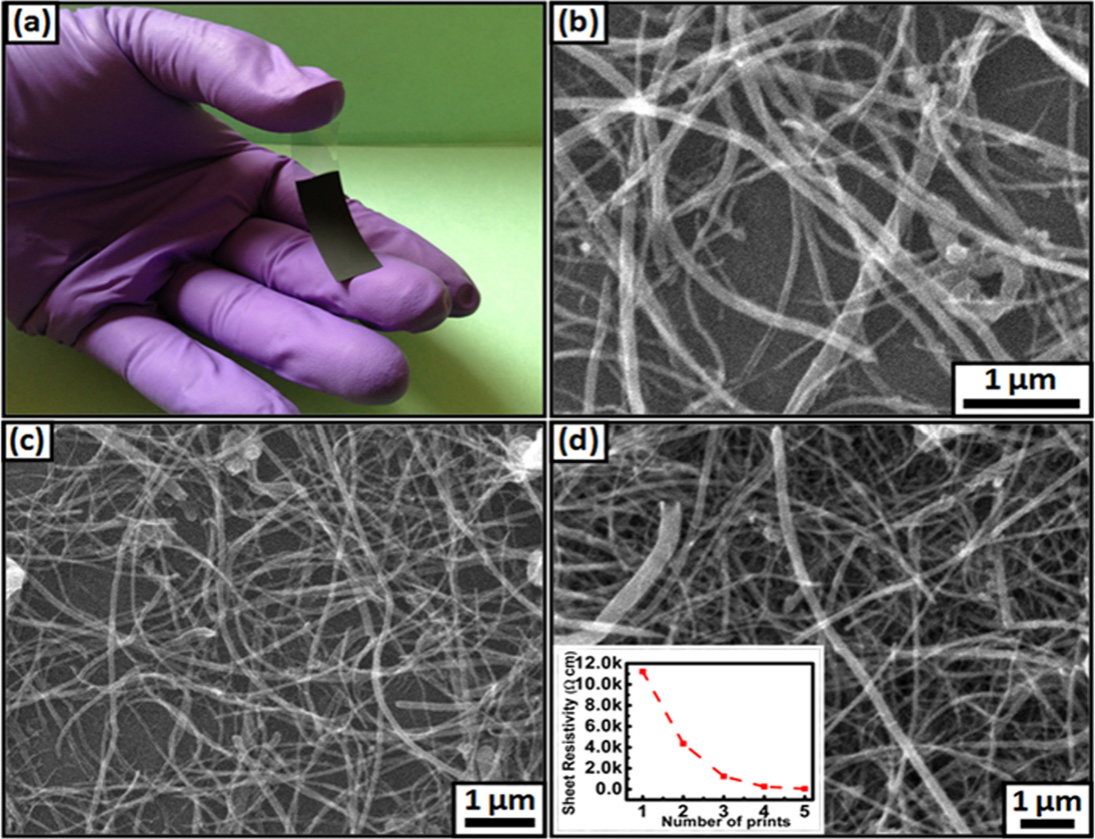
(a) Strips of printed *f*-MWCNT on PET, (b) SEM micrographs of 1 printed layer, (c) 2 printed layers and (d) 5 printed layers. Inset figure shows decrement in resistivity with number of printed layers.

The surface morphology of the inkjet printed films with different printed layers is studied using SEM (Fig 5). It is observed that for single print, CNT are approximately monodispersed on the PET substrate with an average thickness of 35 nm (Fig 5a). However, this monolayer CNTs film exhibits a poor conducting network with resistivity of 11.2 kΩ cm. On increasing the printed layers, the print dots occupy the vacancies or overlap with each other, resulting in the gradual formation of a continuous CNT network with decreased electrical resistance of the CNT film (Fig 5b and 5c). When CNTs are printed for five layers (Fig 5d), the network is dense and covers almost all voids on the film. They form tangled, randomly oriented, and highly conducting networks (thickness ~ 0.2 μm) with a sheet resistivity 65 Ω. cm. The resistivity versus printed layers of *f*-MWCNT is shown in the inset in Fig 5d. As expected, the resistance of the films decreased with the number of print repetitions due to the better percolation between the deposited CNT which can arise due to the presence of the N hetero atom in the cycloaddition of azides MWCNT. These *f*-MWCNT films were used as electrodes for fabricating the supercapacitor and their electrochemical performances were evaluated using CV, EIS, and GCD in neutral, acidic, basic, and organic electrolytes.

### Electrochemical Analysis of *f*-MWCNT//*f*-MWCNT supercapacitor cell (SCs)

**Fig 6:**
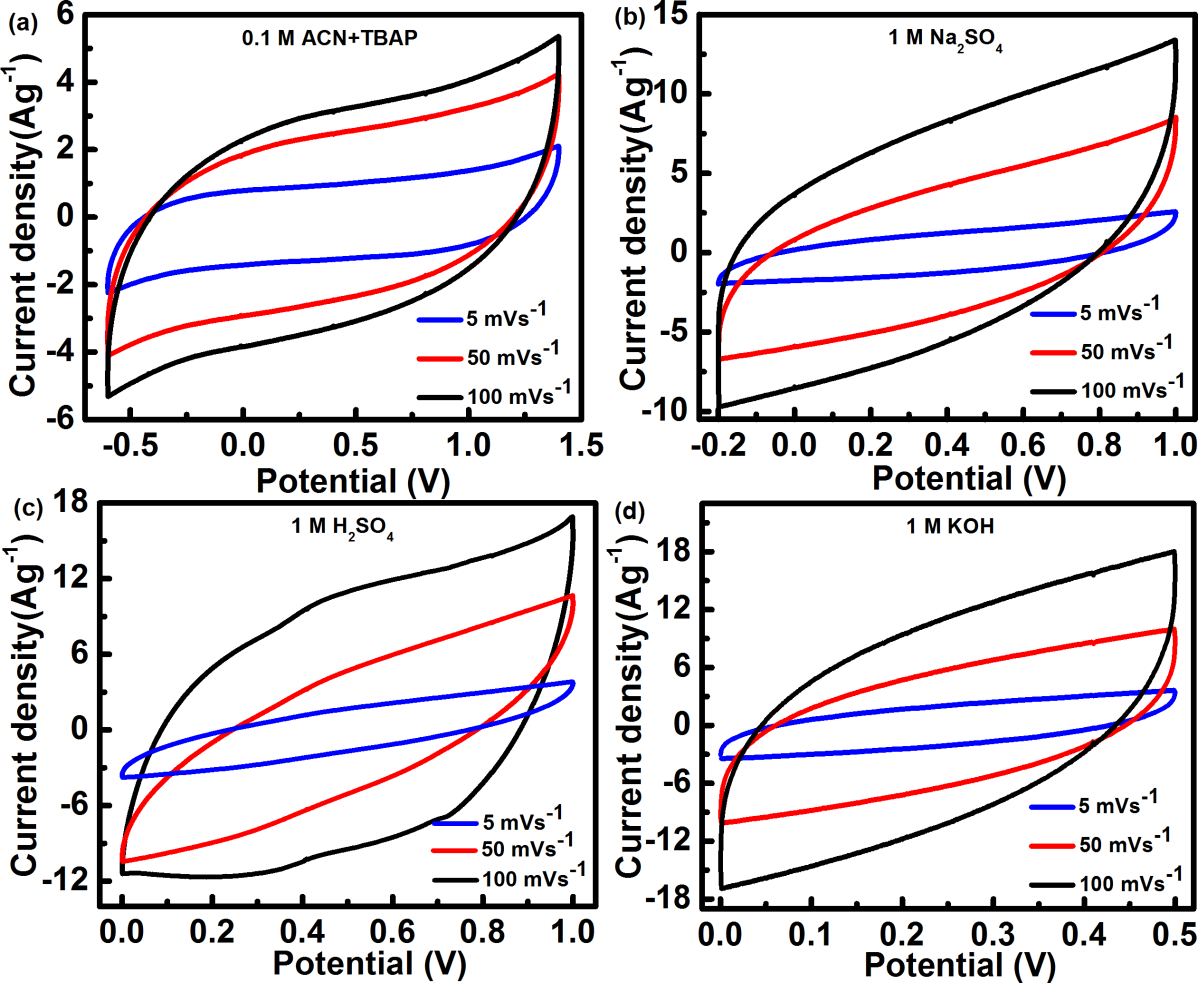
Scan rate dependent Cyclic Voltammograms (CVs) for *f*-MWCNT SCs in different electrolytes.

*f*-MWCNT solubilized in a variety of solvents, can be easily deposited using the printing technique; such MWCNT network based architecture has the potential for fully printable electronics. Moreover, due to the slow kinetics of carbon oxidation, the MWCNT network has a high electrochemical stability compared to amorphous carbon [29]. The electrochemical performance of the *f*-MWCNT SCs was first examined by cyclic voltammetry (CV) by using organic (0.1M ACN+TBAP) and different aqueous electrolytes (1M Na_2_SO_4_, H_2_SO_4_, and KOH). To evade probable interference, the potential sweep range used in experiments was chosen in order to avoid the occurrence of oxidation or reduction of aqueous medium. Fig 6a–6d show the CV result of *f*-MWCNT SC using organic and aqueous solutions in different potential ranges at different scan rates. All CVs maintained a quasi-rectangular shape with a perfect mirror-image feature, even at a high scan rate, suggesting good capacitive behavior and high rate capability of *f*-MWCNT [30,31]. Compared to other carbonaceous materials, *f*-MWCNT exhibits decreased inter-particle resistance and consequently higher conductivity, resulting in improved performance. Moreover, CV of *f*-MWCNT SCs in H_2_SO_4_ demonstrates the occurrence of a reversible couple of peaks due to the oxidation/reduction of surface functional groups represented by

$$-C=O\;\;+\;\;\;\;H^{+}\;\;+\;\;e^{-}\;\;\to \;\;-COH$$

A similar response was also observed in a previous work for carbon nanotubes [32]. Current densities of *f*-MWCNT SCs in aqueous H_2_SO_4_ and KOH were considerably higher than those in organic electrolyte. However, a much larger integrated area in the organic electrolyte compared to that in the aqueous electrolyte indicates higher specific capacitance of ~268 F/g at 5mV/s.

**Fig 7:**
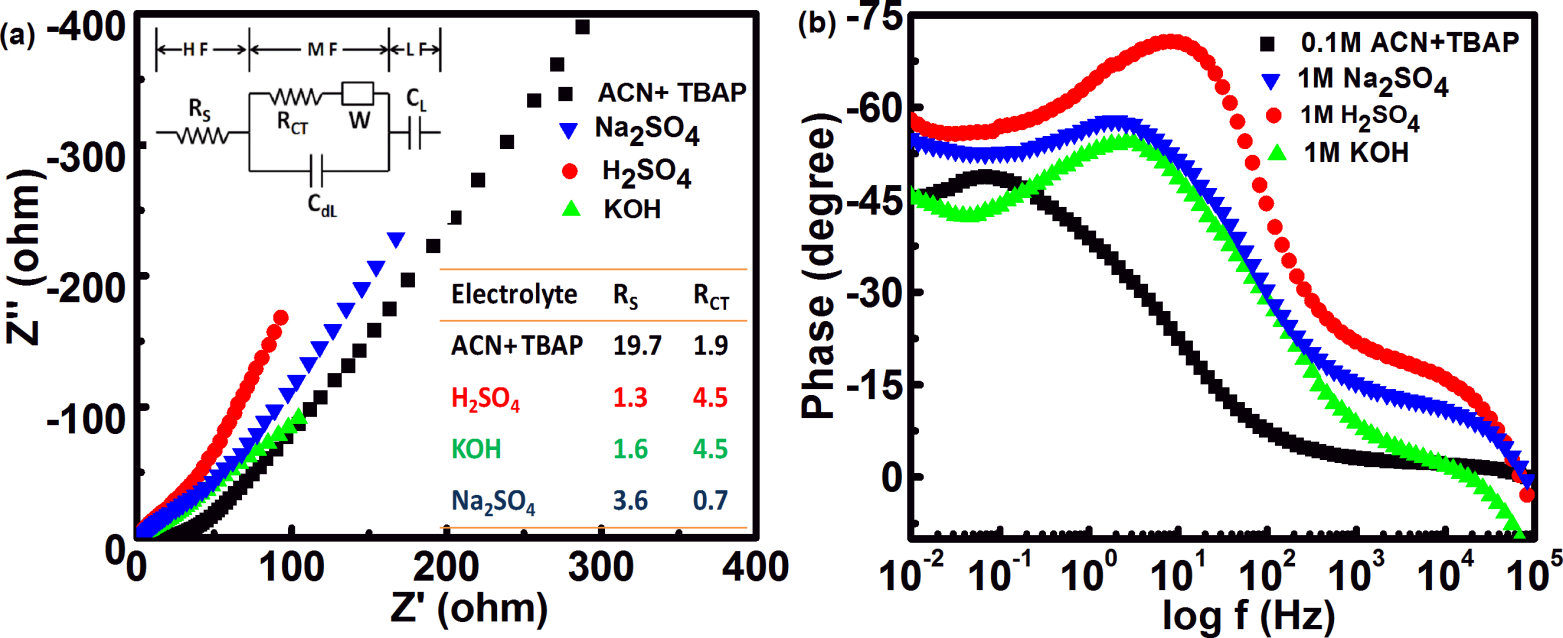
(a) Nyquist plots for *f*-MWCNT SC in different electrolytes. Inset shows equivalent circuit for SC and R_s_/R_CT_ values presented in tabular form. (b) Bode plots of *f*-MWCNT SCs in different electrolytes.

**Fig 8:**
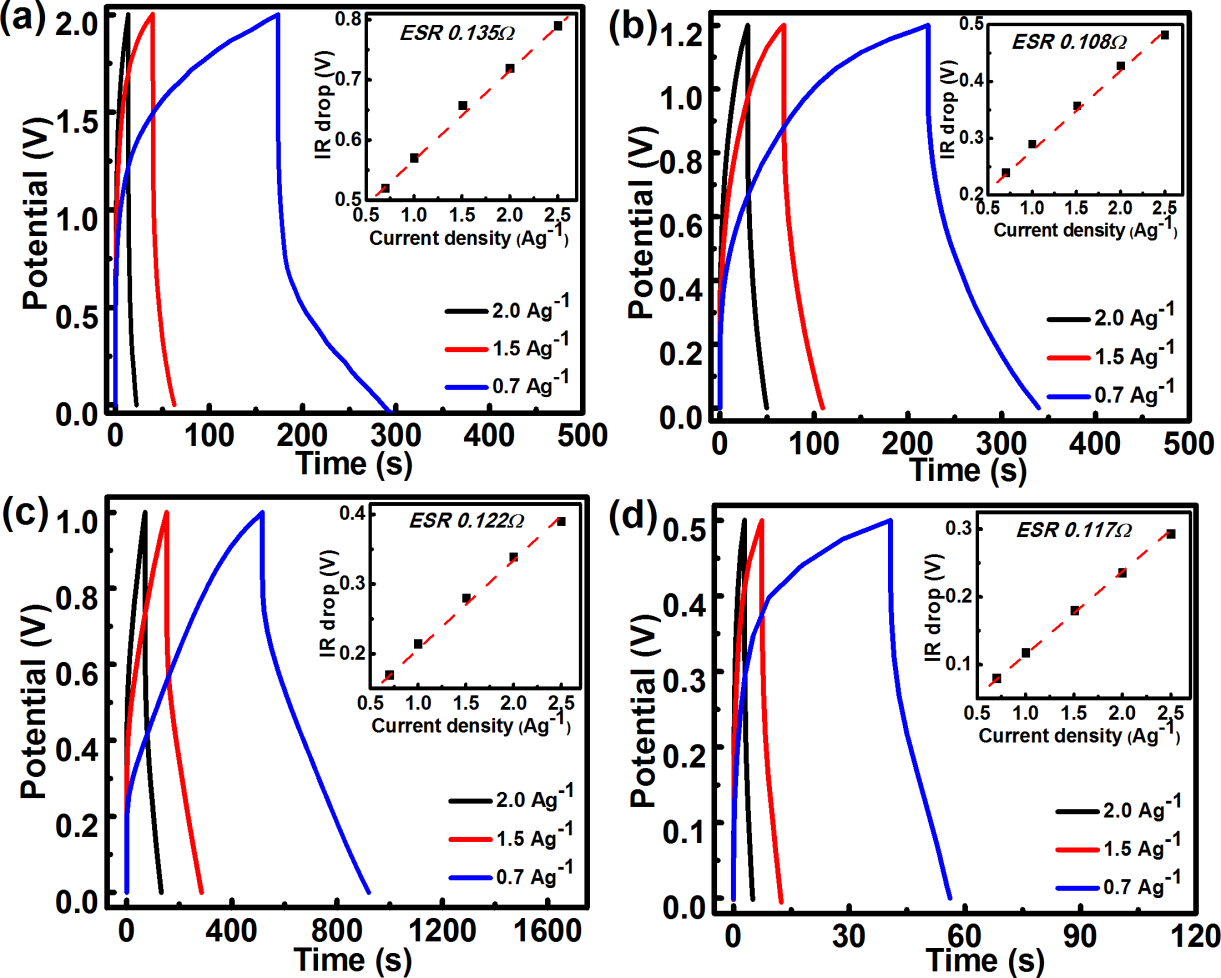
Galvanostatic charge-discharge (GCD) curves of *f*-MWCNT SCs at different current densities in (a) 0.1 M ACN + TBAP, (b) 1 M Na_2_SO_4,_ (c) 1 M H_2_SO_4_, and (d) 1 M KOH. Insets show variation of IR drops at different current densities.

Electrochemical impedance spectroscopy (EIS) was performed to evaluate the frequency response of *f*-MWCNT SCs. Nyquist plot usually exhibits a high-frequency semicircle due to an effective series resistance attributed to the ionic conductivity at the electrode/electrolyte interface. Here, the lack of a semicircle in the high frequency region (Fig 7a) implies a high ionic conductivity at the electrode/electrolyte interface, consistent with the ultrahigh rate capability with high power performance [33]. A Nyquist plot is further fitted with an equivalent circuit (inset) to determine the various resistances, which are tabulated in the second inset. In general, the low frequency region has a capacitive spike parallel to the imaginary axis. It is noted that the low frequency region of *f*-MWCNT SCs in H_2_SO_4_ has relatively improved capacitive character, which is also evident from the high charge storing capacity of the cell. The phase angles of *f*-MWCNT SCs in all electrolytes in the low frequency region (<50 Hz) are close to -90°, indicating a near-ideal capacitive response (Fig 7b) [34], while the SCs using an organic electrolyte show a smaller phase angle. Galvanostatic charge/discharge (GCD) curves of *f*-MWCNT SCs in all the electrolytes are shown in Fig 8a–8d. GCD curves at different current densities show clear deviations from linearity in the charge/discharge profile, indicating the presence of redox processes [32]. The inset shows the relation between the IR drop at different current densities, providing the ESR of the cell in different electrolytes. The lower ESR range from 0.135 to 0.108 ohm is observed in different electrolytes, imparting high power delivery capacity to the SCs [35].

**Fig 9:**
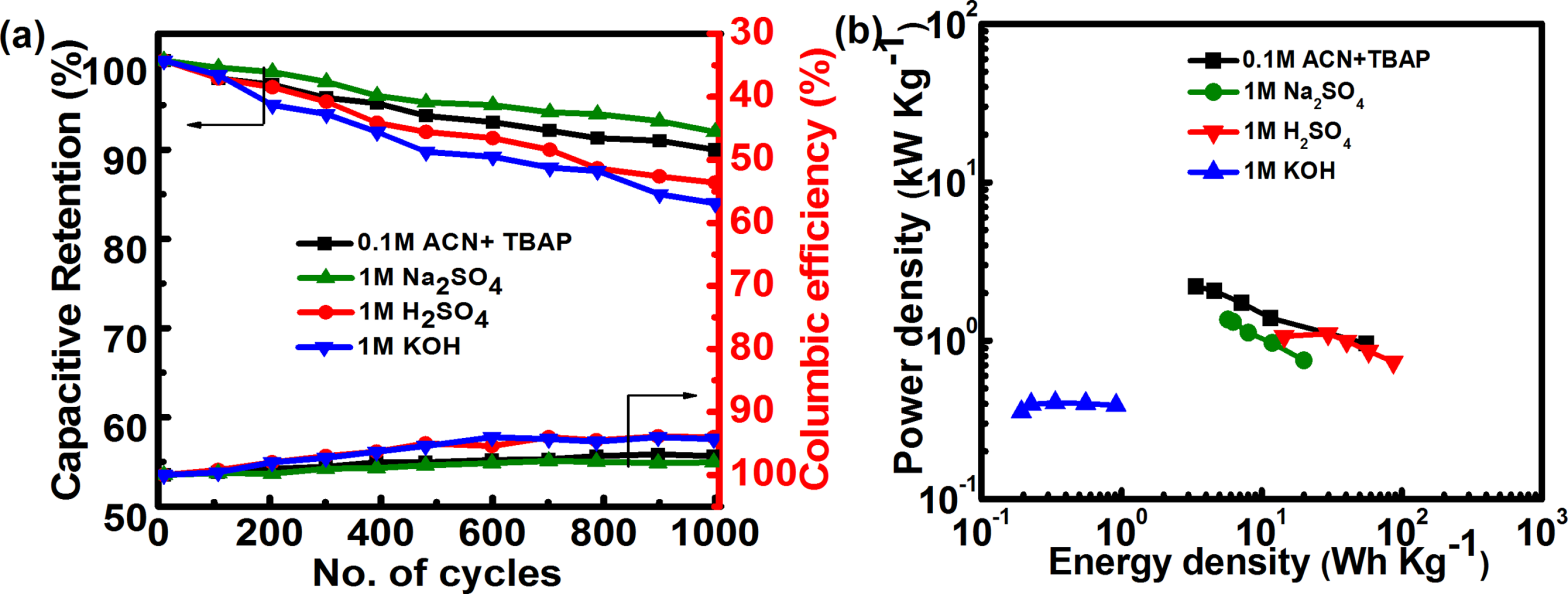
(a) Charge/discharge cycling life test and Columbic efficiency. (b) Ragone plot for *f*-MWCNT SCs in all electrolytes.

Even though redox reactions are generally unfavorable for the long-term stability of supercapacitors as they are associated with self-discharge and leakage current, SCs demonstrate long term stability of the cell of ˃90% in different electrolytes (Fig 9a). High performance of this printable *f*-MWCNT SCs is also reflected in the efficient Columbic efficiency of ≥95% in all the cases. The two major performance indicators of supercapacitors, power density and energy density, are then calculated and shown in a Ragone plot (Fig 9b). It is observed that the power density of the *f*-MWCNT SC in organic electrolyte is significantly higher than that in other electrolytes, while the energy density is higher in 1 M H_2_SO_4_. Furthermore, with the increase in current density from 0.3 to 2 A/g, the power density of the *f*-MWCNT SC in the organic electrolyte increased from 0.9 to 2.2 kW/kg, while the energy density of the *f*-MWCNT SC in 1 M H_2_SO_4_ electrolyte decreased from 86.8 to 14 Wh/kg. Table 1 compares the specific capacitances of the *f*-MWCNT SCs calculated from CV at 5 mV/s with energy and power density in all electrolytes from the discharge curves at 1.5 A/g. The energy and power density are lowest in the KOH electrolyte among all electrolytes due to the lower operational potential of 0.5 V.

**Table 1:** Specific capacitance, energy and power density calculated from CV and GCD curves respectively in different electrolytes.

**Electrolytes**	**C**_**sp**_ **at 5 mV/s (F/g)**	**E.D. at 1.5 A/g (Wh/kg)**	**P.D. at 1.5 A/g (kW/kg)**
****ACN+TBAP****	268 ± 12	4.5 ± 0.2	2 ± 0.09
****H**_**2**_**SO**_**4**_**	240 ± 10	29.7 ± 1.2	1.1 ± 0.05
****Na**_**2**_**SO**_**4**_**	233 ± 9	6.2 ± 0.2	1.2 ± 0.05
****KOH****	120 ± 11	0.22 ± 0.02	0.3 ± 0.03

### Conclusions

We demonstrated a simple method for generating electrically conductive MWCNT with precise control of film thickness on flexible substrate. The presence of carboxylic moieties (-COOH) on azide functionalized MWCNT (*f*-MWCNT) resulted in highly stable aqueous dispersion (max. concentration ~ 10 mg/mL H_2_O), making the suitable for inkjet printing. By applying multiple prints, patterns with a lower sheet resistivity of ~65 Ω cm are successfully achieved. Supercapacitors assembled from these printed electrodes exhibited good electrochemical performance in organic as well as aqueous electrolytes. High specific capacitance of 268 F/g at 5mV/s is achieved in organic electrolyte with the highest energy density of 57.8 Wh/kg at 0.5 A/g in aqueous H_2_SO_4_. Fabricated SC exhibits long term charge/discharge stability with capacitive retention of ~85–94% in different electrolytes. High performance of these SCs is also reflected in the efficient columbic efficiency of ≥95% in all the cases. The efficient functionalization of MWCNT having carboxylic moieties enhances dispersion stability and the presence of nitrogen influences the electronic properties of nanotubes, and hence the device performance.
